# Huanglian-Jie-Du-Tang Extract Ameliorates Depression-Like Behaviors through BDNF-TrkB-CREB Pathway in Rats with Chronic Unpredictable Stress

**DOI:** 10.1155/2017/7903918

**Published:** 2017-06-14

**Authors:** Yi-Lu Ye, Kai Zhong, Dan-Dan Liu, Jing Xu, Bei-Bei Pan, Xiang Li, Yue-Ping Yu, Qi Zhang

**Affiliations:** ^1^Basic Medicine Department, Hangzhou Medical College, Hangzhou 310053, China; ^2^The Second Affiliated Hospital of Zhejiang University School of Medicine, Hangzhou 310009, China

## Abstract

Neuroinflammation is considered as one of the common pathogeneses of depression. Huanglian-Jie-Du-Tang (HJDT) is a traditional Chinese herbal formula. The present study investigates the antidepressant-like effect of HJDT and its possible mechanism in rats. Rats were given HJDT (2, 4, and 8 g/kg, intragastrically), paroxetine (1.8 mg/kg, intragastrically), or an equivalent volume of saline for 42 days. The depression-related behaviors, including sucrose preference test (SPT), open field test (OFT), novel objective recognition task (NORT), and forced swimming test (FST), were detected. 5-Hydroxytryptamine (5-HT) and dopamine (DA) contents, microglial activation, proinflammatory cytokines, and brain derived neurotrophic factor (BDNF), tropomyosin receptor kinases B (TrkB), and cAMP-responsive element binding protein (CREB) expression were investigated. The results indicated HJDT (2 and 4 g/kg) dramatically ameliorated the depression-like behaviors. Also HJDT decreased the number of microglia and the proinflammatory cytokines in hippocampus. Western-blotting analysis displayed HJDT upregulated BDNF, TrkB, and pCREB/CREB expression in hippocampus. Particularly, pCREB DNA activity enhanced with HJDT treatment in hippocampus. But there was no difference in the 5-HT and DA contents with HJDT treatment. In conclusion, it was supposed that HJDT might be a potential Chinese medicine decoction for treating or alleviating complex symptoms of depression through BDNF-TrkB-CREB pathway.

## 1. Introduction

Depression is a serious mental disorder with a high prevalence worldwide and causes serious consequences for the individual's quality of life [1]. At present most effective antidepressants to treat depression target serotonin and/or norephinepherine. However, many of these drugs have a slow onset of action (over 2 weeks) and are effective in only 50% of patients, with serious adverse reactions [2]. Therefore, it is necessary to explore a more powerful and safer antidepressants, which may influence different targets. Traditional Chinese Medicine (TCM) has been commonly considered effective and safe, including many herbal formulae with psychotropic potential.

Huanglian-Jie-Du-Tang (HJDT) is a traditional Chinese herbal formula composed of the roots of* Coptis chinensis *Franch.,* Scutellaria baicalensis *Georgi,* Phellodendron chinense *Schneid., and the fruit of* Gardenia jasminoides *Ellis, which is widely used in China, Japan, and Korea. It was recorded in the traditional Chinese medicinal book* Wai-Tai-Mi-Yao*. At present it is widely used for alleviating the symptoms of gastrointestinal issues [3], liver damage [4], Alzheimer's disease [5], ischemic brain injury [6, 7], and so forth. In our previous study, the HPLC results demonstrated that the main active components of HJDT were berberine, baicalin, wogonoside, and gardenoside [7], all of which had been found with antidepressant effect. Berberine could attenuate depression- and anxiety-like behaviors following chronic morphine withdrawal in rats [8]. By normalizing glucocorticoid receptor (GR) function through SGK1- and FKBP5-mediated GR phosphorylation, baicalin also improved the depression-like behaviors induced by chronic mild stress in mice [9]. So it was speculated that HJDT could have the basis on anxiolytic and antidepressant effects.

Microglia are important resident immunoreactive cells in the central nervous system (CNS). Commonly, microglia were considered to be “resting,” becoming “activated” upon allostatic changes to coordinate immune-like responses [10] and triggering inflammation reactions through chemokine and cytokine [11]. The available evidence indicates that depression is tightly associated with alterations of microglia and inflammation [12], which manifested with more proinflammatory profile [13] and decreased microglia-derived brain derived neurotrophic factor (BDNF) [14]. BDNF and its receptor tropomyosin receptor kinases B (TrkB) as well as its downstream target cAMP-responsive element binding protein (CREB) played crucial role in depression [15, 16]. Therefore this study examined the effects of HJDT on the depression-like behaviors and potential mechanism involved in inflammatory reaction and BDNF-TrkB-CREB pathway.

## 2. Materials and Methods

### 2.1. Plant Materials and HJDT Preparation

The roots of* Coptis chinensis *Franch. (141107, Sichuan, China),* Scutellaria baicalensis *Georgi (150113, Neimenggu, China),* Phellodendron chinense *Schneid. (150203, Sichuan, China), and the fruit of* Gardenia jasminoides *Ellis (140926, Jiangxi, China) were purchased from Eastern China Medical Corp. (Zhejiang Province, China) and identified as being in families Ranunculaceae, Labiatae, Rutaceae, and Rubiaceae, respectively. Then they were authenticated by Professor H. X., Sun (College of Animal Sciences, Zhejiang University). According to our previous report, aqueous extract of HJDT was prepared [7]. The ultima concentration was 1 g/mL.

### 2.2. Active Components of HJDT Determination

The HPLC was carried out as described in our previous study [7]. Briefly, HPLC conditions are as follows: eluent A, acetonitrile; eluent B, 0.05% potassium dihydrogen phosphate and 0.05% triethanolamine-water (pH 3.0 adjusted by phosphoric acid); gradient, 0 min (A–B, 14 : 86) → 12 min (A–B, 25 : 75) → 27 min (A–B, 30 : 70) → 28 min (A–B, 14 : 86); and then it was equilibrated with A–B (14 : 86) for 12 min at a flow of 0.8 mL/min. The contents of the analytics were determined from the corresponding calibration curves.

### 2.3. Animals

Male Sprague-Dawley rats (Shanghai Slyke Laboratory Animal Limited Corporation, Certificate number SCXK 2012-0002, China) were handled in accordance with the National Institute of Health Guide for the Care and Use of Laboratory Animals and the norms of the Ethics Committee for Animal Research of Hangzhou Medical College. They were housed under a 12 : 12 h light/dark cycle, controlled temperature of 22 ± 1°C, and humidity of 50 ± 10%. Food and water were provided ad libitum.

### 2.4. Groups and Drug Treatments

After environmental adaption for one week, the rats were assigned to one of the following groups according to their sucrose preference ratio and body weight: normal control group (only saline treatment and no chronic unpredictable stress (CUS)); CUS depression group (saline treatment and CUS); paroxetine group (1.8 mg/kg, GlaxoSmithKline, England, paroxetine treatment group and CUS); HJDT groups (2, 4, and 8 g/kg, HJDT treatment and CUS). Rats were given intragastrically either paroxetine, HJDT, or saline for 42 days after the CUS, and at 1 h before the beginning of the CUS procedure or behavioral tests. The dose of paroxetine and HJDT were chosen based on the previous reports [7, 17].

### 2.5. Experimental Protocol

**Figure 1:**
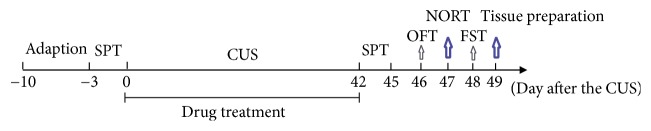
Schedule of the experimental protocol.

Except normal control group, rats in other groups were subjected to two stressors on an unpredictable order and at an unpredictable time each day following gavage for 6 weeks. The stresses included food deprivation for 24 h, water deprivation for 24 h, inversion of day/night cycle, forced swimming for 6 min, 45° tilted cage for 7 h, soiled cage bedding overnight, and restraint for 5 min. Rats in normal control group were left undisturbed during the period. The body weight was measured once weekly from day 0 after the CUS. Sucrose preference test (SPT), open field test (OFT), novel objective recognition task (NORT), and forced swimming test (FST) were performed from day 42 to day 48 after the CUS, respectively (see Figure 1).

### 2.6. Behavioral Tests

#### 2.6.1. Sucrose Preference Test (SPT)

The SPT was carried out as previous report with slight modification [18]. The volumes of sucrose solution and water consumed were recorded at the 4th day during SPT. At that day, rats were housed in individual cages and free to access two bottles containing 100 mL of sucrose solution (1% w/v) and 100 mL of water, respectively. After 3 h of exposure, the volumes of sucrose solution and water consumed were recorded. At last, the sucrose consumption ratio was calculated: sucrose solution consumption ratio (%) = [sucrose solution consumption volume/(water consumption volume + sucrose solution consumption volume)] × 100.

#### 2.6.2. Open Field Test (OFT)

Each rat was placed gently into the center of a brightly lit open field arena (80 × 80 × 40 cm). The locomotor tracks were continuously recorded by a video camera for 5 min and analyzed by a computerized video tracking system (EthoVision XT, Noldus, Netherlands). The following parameters were determined: (a) total traveled distance (m), (b) time in central region (s), (c) traveled central distance ratio, and (d) rearing number. After each test session, the enclosures were thoroughly cleaned with 70% ethanol solution.

#### 2.6.3. Novel Object Recognition Task (NORT)

The task consisted of three phases: T_1_ phase, interval time, and T_2_ phase. During habituation, rats were introduced in the empty chamber and left to freely explore it for 10 min. Afterwards, they were put into their home cages for 5 min, during which two identical objects (familiar object: cubic crude wood with 4 cm diameter) were placed in the chamber oppositely. In the T_1_ phase, rats explored the objects for 5 min. After a 1.5 h interval time, rats were placed into the chamber again for 5 min, when one of the familiar objects was replaced by a novel one (novel object: cylindrical crude wood with 4 cm diameter and height), that is, T_2_ phase. Tracks were recorded and analyzed by a computerized video analyzer (EthoVision XT, Noldus, Netherlands). The following parameters were analyzed: total exploration time to familiar and novel objects, discrimination ratio (DR, DR = [*N*/(*N* + *F*)] × 100%), and discrimination index (DI, DI = [(*N* − *F*)/(*N* + *F*)] × 100%). *N* is exploration time with the novel object (min); *F* is exploration time with the familiar object (min).

#### 2.6.4. Forced Swimming Test (FST)

One day before proceeding for the test, rats were trained for swimming for 15 min in a glass cylinder (60 cm height × 25 cm diameter) filled with water at 30 cm height and under temperature 25 ± 2°C. At the next day, rats were individually subjected to swimming for 6 min of which initial 2 min was for acclimatization. And the immobility time during the last 4 min was recorded.

### 2.7. 5-Hydroxytryptamine (5-HT) and Dopamine (DA) Detection

Rats were decapitated and the hippocampus was separated on ice quickly at day 49 after the CUS. Then it was sonicated to obtain tissue homogenates. After removing particulars by centrifugation (2000 ×g, 4°C, 20 min), assay was immediately detected. 5-HT (number EIA-3325) and DA (number EIA-3236) were measured using respective ELISA system (Shanghai ELISA Biotech Co., Ltd, China) according to the manufacturer's instructions. Finally, optical density was determined (absorbance at 450 nm) on a plate reader.

### 2.8. Brain Cryosections Preparation and Microglia Number Measurement

Rats were anesthetized after the behavioral tests at day 49. To investigate the activation and the number of microglia in the hippocampal CA1 region and DG, immunohistochemical methods were performed with the polyclonal rabbit antibodies against Iba-1 (1 : 200, Wako, Japan, number 019-19741). The brain cryosections preparation and immunohistochemical methods were based on our previous report (Ye et al., 2016). Normal goat serum was used instead of the primary antibody in the control sections. The number of positive cells in the hippocampal CA1 region and DG was calculated with three different fields.

### 2.9. Proinflammatory Cytokines Concentration Measurement

Total RNA was extracted from hippocampus using Trizol reagent (Invitrogen Life Technologies, USA). For cDNA synthesis, 1.5 *μ*g RNA was reversely transcribed with superscript™ III reverse transcriptase (Invitrogen Life Technologies, USA). Semiquantitative PCR was performed using primers specific for TNF-*α*, iNOS, IL-1*β*, IL-6, IL-10, and GAPDH. The primers (Shanghai Yingjun Biotechnology Co., Ltd, China) were as follows: 

**Table:** 

TNF-*α*	F: 5′CTGGCGTGTTCATCCGTTCT3′	R: 5′GCCACTACTTCAGCGTCTCGT3′
iNOS	F: 5′TTGGAGCGAGTTGTGGATTG3′	R: 5′TGAGGGCTTGCCTGAGTGA3′
IL-1*β*	F: 5′CCTCTGACAGGCAACCACTTA3′	R: 5′GCACTGGTCCAAATTCAATTC3′
IL-6	F: 5′TGCCTTCTTGGGACTGATGT3′	R: 5′ATACTGGTCTGTTGTGGGTGGT3′
IL-10	F: 5′TTGAACCACCCGGCATCTAC3′	R: 5′CCAAGGAGTTGCTCCCGTTA3′
GAPDH	F: 5′GGAAAGCTGTGGCGTGAT3′	R: 5′AAGGTGGAAGAATGGGAGTT3′

The products of RT-PCR were separated by electrophoresis using 1.2% agarose gel and stained with ethidium bromide. Densitometric analysis was performed using image analysis software.

### 2.10. BDNF, TrkB, CREB, and pCREB Expression Measurement

Samples of hippocampus were homogenized on ice in PBS containing 1% protease inhibitor and 1% phosphatase inhibitor for Western-blotting. Proteins were obtained by centrifugation at 14000 rpm at 4°C for 15 min and quantified by Bradford assay (BioRad, USA). A 50 *μ*g sample of each group was subjected to electrophoresis using 20% SDS at 80 V. The proteins were transferred to polyvinylidene fluoride membranes at 250 mA for 2 h. Antibodies for BDNF (1 : 1000, Santa Cruz Biotechnology, USA, number sc-546), TrkB (1 : 1000, Santa Cruz Biotechnology, USA, number sc-8316), CREB (1 : 2000, Cell Signaling Technology, USA, number 9197), pCREB (1 : 2000, Cell Signaling Technology, USA, number 9198), and GAPDH (1 : 10000, KangChen Bio-Tech, Shanghai, China) were applied overnight. Then membranes were incubated with horseradish peroxidase (HRP) secondary antibody (KangChen Bio-Tech, Shanghai, China). At last, the signal intensities of proteins were analyzed using Image J software.

### 2.11. pCREB DNA Activity Detection

The TransAM assay for pCREB activity (Active Motif, USA, number 42096) was performed according to the manufacturer's instructions. Briefly, oligonucleotides containing the pCREB consensus binding site were immobilized on a 96-well plate. The active forms of pCREB in the nuclear extracts were bound to the oligonucleotides on the plate and detected colorimetrically. The samples were measured at an absorbance of 450 nm on a spectrophotometer.

### 2.12. Statistical Analysis

All of the data were expressed as mean ± SEM. Repeated measures of one-way analysis of variance tests (ANOVA) and Fisher's least significant differences (LSD) post hoc analysis tests were performed to analyze the significance of any treatment effect among the groups by SPPS 16.0 for Windows except the effects of HJDT on NORT. *t*-test was used to compare the difference of DR and DI between T_2_ phase and T_1_ phase, the exploration time to novel and familiar objects. *p* < 0.05 was considered as statistical significance.

## 3. Results

### 3.1. The Content of Active Components in HJDT

Using HPLC, we detected the contents of 7 active components in the decoction of HJDT (Table 1). The results showed the content of baicalin, berberine, wogonoside, and geniposide was higher than others, which was demonstrated as 5.23%, 4.25%, 1.5%, and 1.21%, respectively. They may be the main active components of HJDT for depression.

### 3.2. HJDT Had No Effect on the Body Weight Decrease after the CUS

**Figure 2:**
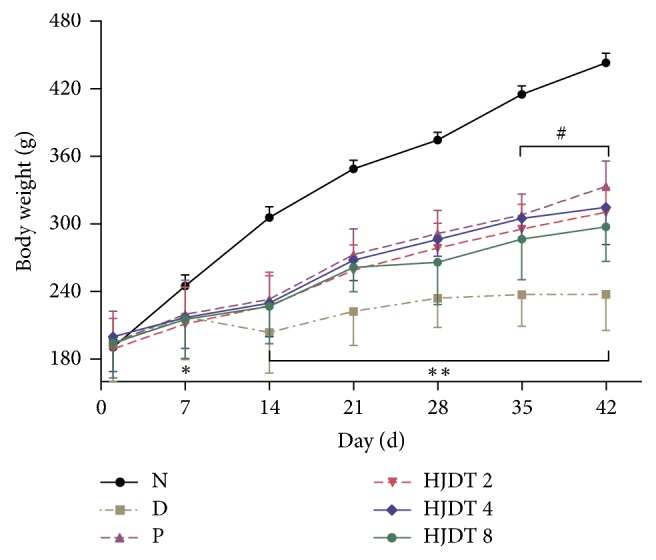
Effects of HJDT on the body weight after the CUS in rats. The body weight of rats in CUS depression group decreased from day 7 to day 42 after the CUS. Paroxetine group gained the body weight from day 35 after the CUS. HJDT had no effect on the body weight decrease after the CUS. ^*∗*^*p* < 0.05 and ^*∗∗*^*p* < 0.01 relative to the normal control group; ^#^*p* < 0.05 relative to the CUS depression group. N: normal control group; D: CUS depression group; P: paroxetine group; HJDT 2: HJDT 2 g/kg group; HJDT 4: HJDT 4 g/kg group; HJDT 8: HJDT 8 g/kg group.

As shown in Figure 2, all groups were equivalent in their body weight prior to the onset of CUS. The body weight in the normal control group gradually increased from day 7 to day 42 after the CUS. Compared to the normal control group, rats exposed to the CUS showed significantly lower body weight from day 7 to day 42 after the CUS. It showed a significant body weight gain after chronic treatment with paroxetine from day 35 to day 42 after the CUS, whereas HJDT had no effect on it, compared to the CUS depression group.

### 3.3. HJDT Increased the Sucrose Consumption Ratio in SPT after the CUS

**Figure 3:**
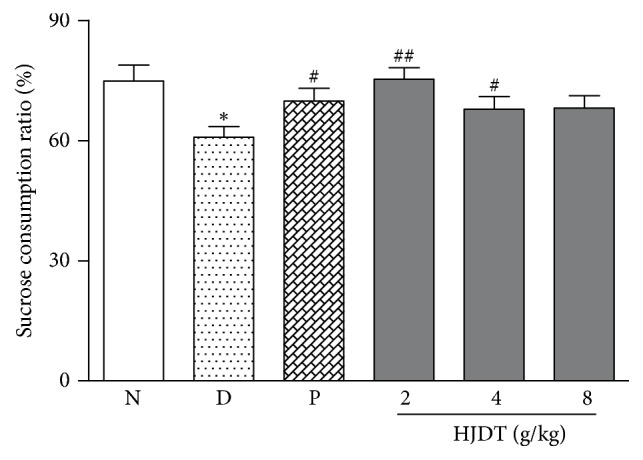
The effect of HJDT on the sucrose consumption ratio in SPT after the CUS. The sucrose consumption ratio decreased in the CUS depression group. Treatment with paroxetine and HJDT (2, 4 g/kg) increased the sucrose consumption ratio. ^*∗*^*p* < 0.05 relative to the normal control group; ^#^*p* < 0.05 and ^##^*p* < 0.01 relative to the CUS depression group. N: normal control group; D: CUS depression group; P: paroxetine group; HJDT 2: HJDT 2 g/kg group; HJDT 4: HJDT 4 g/kg group; HJDT 8: HJDT 8 g/kg group.

Prior to the onset of CUS, all experimental groups showed similar sucrose consumption ratio (data not shown). But at day 43 after the CUS, CUS depression group showed a significant decrease of sucrose consumption ratio, compared with the normal control group (Figure 3). Paroxetine and HJDT (2 and 4 g/kg) treatment reversed the decrease of sucrose consumption ratio at day 43 after the CUS. However, HJDT 8 g/kg had no effect on the sucrose consumption ratio, compared with the CUS depression group.

### 3.4. HJDT Improved the Locomotor Activity in OFT after the CUS

**Figure 4:**
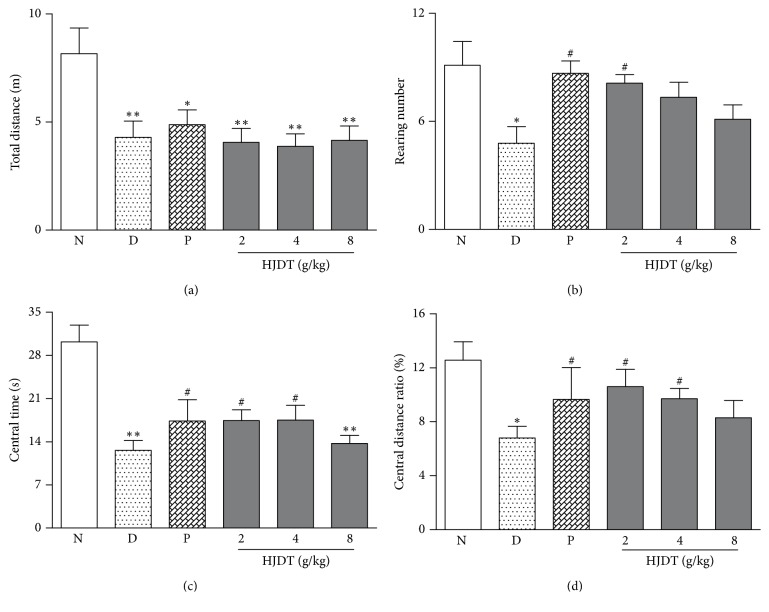
Effects of HJDT on the locomotor activity in OFT after the CUS. Traveled total distance decreased in all rats when exposed to CUS (a). The rearing number decreased in CUS depression group and was reversed with paroxetine and HJDT 2 g/kg treatment (b). The central time and central distance ratio both decreased in CUS depression group and reversed with paroxetine and HJDT (2, 4 g/kg) treatment (c, d). ^*∗*^*p* < 0.05 and ^*∗∗*^*p* < 0.05 relative to the normal control group; ^#^*p* < 0.05 relative to the CUS depression group. N: normal control group; D: CUS depression group; P: paroxetine group; HJDT 2: HJDT 2 g/kg group; HJDT 4: HJDT 4 g/kg group; HJDT 8: HJDT 8 g/kg group.

After the CUS, the total traveled distance in experimental groups, exposed to the CUS, decreased in OFT as compared with the normal control group (Figure 4(a)). The results in Figures 4(b)–4(d) showed that CUS depression rats decreased the rearing number, central time, and central distance ratio in OFT. As expected, paroxetine and HJDT 2 g/kg treatment increased the rearing number, whereas there was no effect in rats treated with HJDT 4 and HJDT 8 g/kg (Figure 4(b)). Also one-way ANOVA revealed that rats treated with paroxetine and HJDT (2 and 4 g/kg) increased the central time and central distance ratio in OFT while HJDT 8 g/kg had no effects (Figures 4(c) and 4(d)).

### 3.5. HJDT Improved the Cognitive Decline in NORT after the CUS

**Figure 5:**
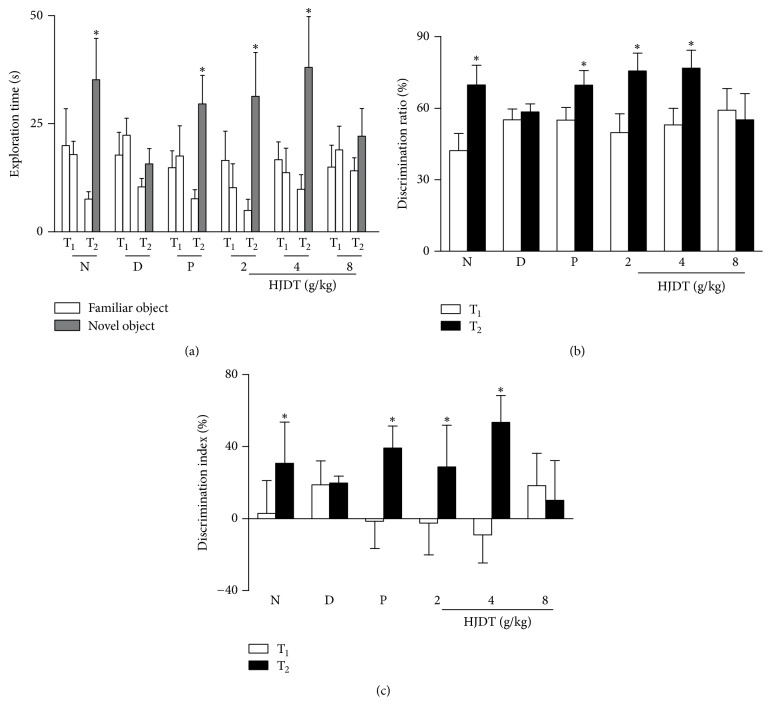
Effects of HJDT on the exploration time, DR, and DI in NORT after the CUS. The exploration time to novel object increased compared with familiar object in T_2_ phase in normal control group, paroxetine group, and HJDT (2, 4 g/kg) groups (a). DR (b) and DI (c) in T_2_ phase significantly increased in normal control group, paroxetine group, and HJDT (2, 4 g/kg) groups compared to T_1_ phase. ^*∗*^*p* < 0.05 relative to the familiar object in T_2_ test (a); ^*∗*^*p* < 0.05 relative to T_1_ phase in the same group (b-c). N: normal control group; D: CUS depression group; P: paroxetine group; HJDT 2: HJDT 2 g/kg group; HJDT 4: HJDT 4 g/kg group; HJDT 8: HJDT 8 g/kg group.

As shown in Figure 5(a), there was no difference in the exploration time in T_1_ phase among groups after the CUS. However, in T_2_ phase, the exploration time to the novel object was longer than to the familiar object in the normal control group, paroxetine group, and HJDT (2 and 4 g/kg) groups. CUS depression group and HJDT 8 g/kg group had no difference in the exploration time between the familiar object and the novel object in T_2_ phase. Figures 5(b) and 5(c) showed there were no differences in the DR and the DI in T_1_ phase among all groups after the CUS. However, the DR and the DI in T_2_ phase in normal control group, paroxetine group, and HJDT (2 and 4 g/kg) groups showed significant increase (Figures 5(b) and 5(c)). CUS depression group and HJDT 8 g/kg group had no differences in the DR and the DI in T_2_ phase when compared to T_1_ phase. It suggested that HJDT (2 and 4 g/kg) and paroxetine could improve the cognitive decline induced by CUS.

### 3.6. HJDT Decreased the Immobility Time in FST after the CUS

**Figure 6:**
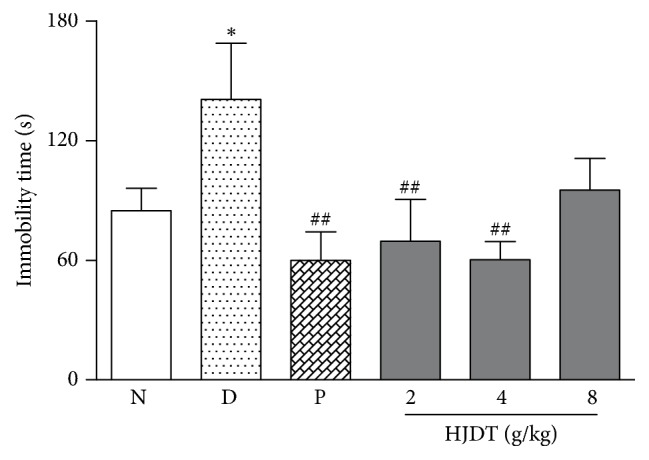
Effect of HJDT on the immobility time in FST after the CUS. The immobility time increased in CUS depression group and was reversed after treatment with paroxetine and HJDT (2, 4 g/kg). ^*∗*^*p* < 0.05 relative to the normal control group; ^##^*p* < 0.01 relative to the CUS depression group. N: normal control group; D: CUS depression group; P: paroxetine group; HJDT 2: HJDT 2 g/kg group; HJDT 4: HJDT 4 g/kg group; HJDT 8: HJDT 8 g/kg group.

The immobility time increased in the CUS depression group compared with the normal control group (Figure 6). HJDT (2 and 4 g/kg) and paroxetine treatment reversed the increase of immobility time induced by the CUS.

### 3.7. HJDT Decreased the Microglia Number in the Hippocampal CA1 Region and DG

**Figure 7:**
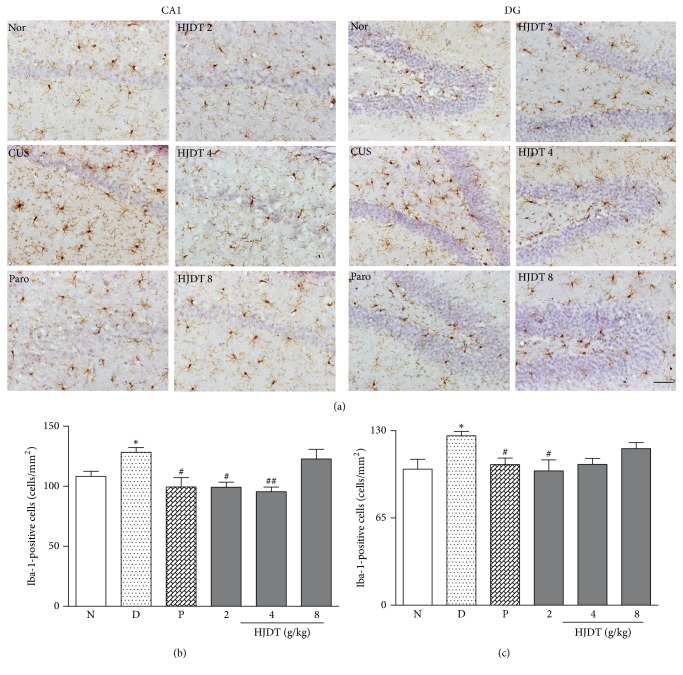
Effect of HJDT on the microglia morphology and number in the hippocampal CA1 region and DG after the CUS. The representative morphology and the number change of microglia in all groups (a). The number of Iba-1 positive cells in hippocampal CA1 region increased in CUS depression group and was reversed with paroxetine and HJDT (2, 4 g/kg) treatments (b). The number of Iba-1 positive cells in hippocampus DG increased in CUS depression group and was reversed with paroxetine and HJDT 2 g/kg treatment (c). ^*∗*^*p* < 0.05 relative to the normal control group; ^#^*p* < 0.05 and ^##^*p* < 0.01 relative to the CUS depression group. Nor/N: normal control group; CUS/D: CUS depression group; Paro/P: paroxetine group; HJDT 2: HJDT 2 g/kg group; HJDT 4: HJDT 4 g/kg group; HJDT 8: HJDT 8 g/kg group. Scale bar = 100 *μ*m, ×200.

Iba-1 positive cell represents the activated microglia. The results of immunohistochemistry detection revealed the morphological and number change of microglia (Figure 7(a)). Figures 7(b) and 7(c) showed the microglia number increased in both hippocampal CA1 region and DG after the CUS. Treatment with paroxetine and HJDT 2 g/kg decreased the microglia number in hippocampal CA1 region and DG (Figures 7(b) and 7(c)). HJDT 4 g/kg decreased microglia number only in hippocampal CA1 region, but not in DG (Figure 7(b)). However, HJDT 8 g/kg had no effect on the microglia number in both the hippocampal CA1 region and DG (Figures 7(b) and 7(c)).

### 3.8. HJDT Increased the BDNF, TrkB, and pCREB/CREB Expression in the Hippocampus after the CUS

**Figure 8:**
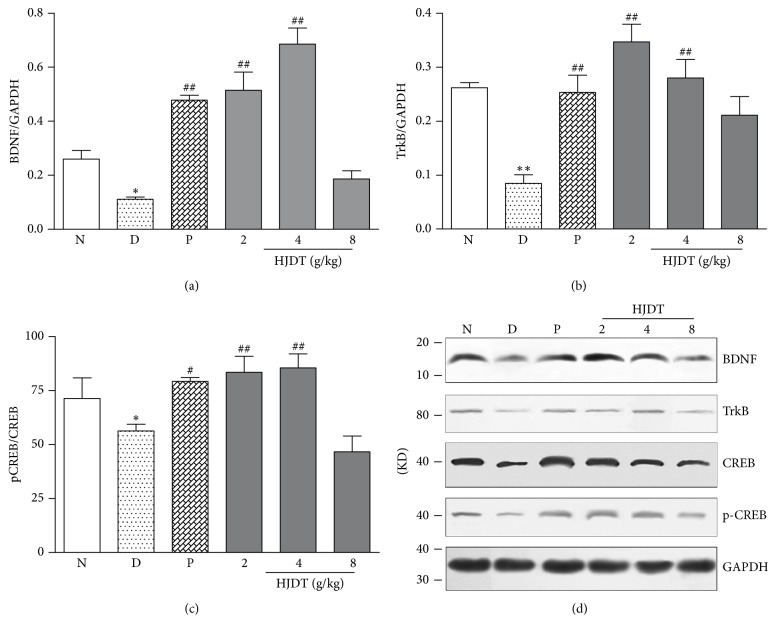
Effect of HJDT on the BDNF, TrkB, and pCREB/CREB expression in the hippocampus after the CUS. The expression of BDNF (a), TrkB (b), and pCREB/CREB (c) decreased in the CUS depression group and was reversed with paroxetine and HJDT (2, 4 g/kg) treatment. Protein expression is induced by CUS and drug treatment in the hippocampus (d). ^*∗*^*p* < 0.05 and ^*∗∗*^*p* < 0.01 relative to the normal control group; ^#^*p* < 0.05 and ^##^*p* < 0.01 relative to the CUS depression group. N: normal control group; D: CUS depression group; P: paroxetine group; HJDT 2: HJDT 2 g/kg group; HJDT 4: HJDT 4 g/kg group; HJDT 8: HJDT 8 g/kg group.

BDNF, TrkB, CREB, and pCREB protein expression was detected by Western-blotting method (Figure 8(d)). Results showed the BDNF, TrkB, and pCREB/CREB expression decreased in the CUS depression group, compared with the normal control group (Figures 8(a)–8(c)). After treatment with paroxetine or HJDT (2 and 4 g/kg), BDNF, TrkB, and pCREB/CREB expression increased (Figures 8(a)–8(c)).

### 3.9. HJDT Enhanced the pCREB DNA Activity in the Hippocampus

**Figure 9:**
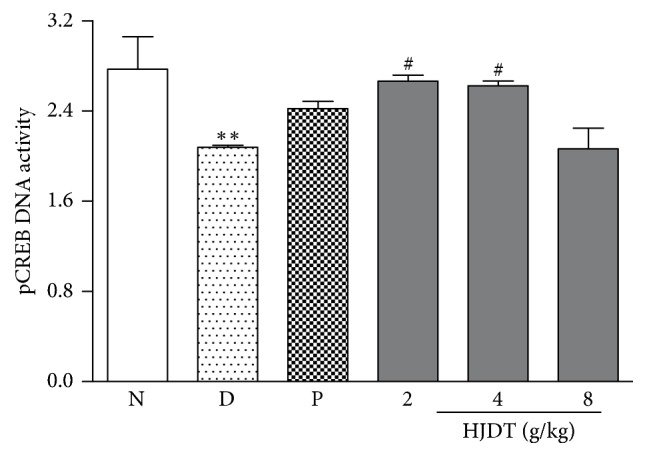
Effect of HJDT on the pCREB DNA activity in the hippocampus after the CUS. The pCREB DNA activity decreased in the hippocampus and was reversed with HJDT (2, 4 g/kg) treatment and there was no effect with paroxetine treatment. ^*∗∗*^*p* < 0.01 relative to the normal control group; ^#^*p* < 0.05 relative to the CUS depression group. N: normal control group; D: CUS depression group; P: paroxetine group; HJDT 2: HJDT 2 g/kg group; HJDT 4: HJDT 4 g/kg group; HJDT 8: HJDT 8 g/kg group.

The pCREB OD_593_ value in the normal control group was 2.77 ± 0.09. But in the CUS depression group, it decreased to 2.08 ± 0.03. After treatment with HJDT (2 and 4 g/kg), it increased to 2.66 ± 0.10 and 2.63 ± 0.09, respectively (Figure 9). It suggested the pCREB DNA activity improved. Compared with the CUS depression group, paroxetine and HJDT 8 g/kg did not affect the pCREB DNA activity (Figure 9).

### 3.10. HJDT Decreased the Concentration of TNF-*α*, iNOS, and IL-6/IL-10 Ratio in the Hippocampus

**Figure 10:**
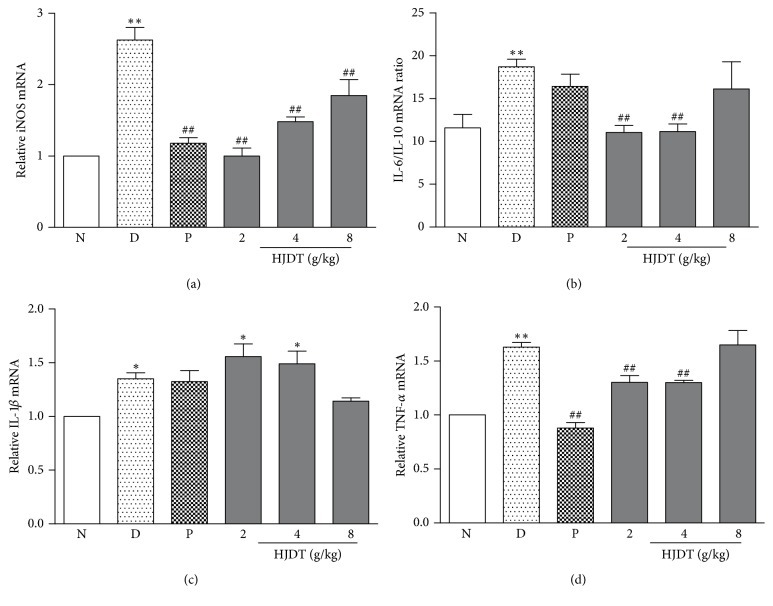
Effect of HJDT on proinflammatory and anti-inflammatory cytokines mRNA in the hippocampus after the CUS. The effects of HJDT on the iNOS mRNA (a), IL-6/IL-10 mRNA ratio (b), IL-1*β* mRNA (c), and TNF-*α* mRNA (d) in the hippocampus. ^*∗*^*p* < 0.05 and ^*∗∗*^*p* < 0.01 relative to the normal control group; ^##^*p* < 0.01 relative to the CUS depression group. N: normal control group; D: CUS depression group; P: paroxetine group; HJDT 2: HJDT 2 g/kg group; HJDT 4: HJDT 4 g/kg group; HJDT 8: HJDT 8 g/kg group.

The results of RT-PCR showed that CUS depression model was related with inflammation reaction. The mRNA of proinflammatory cytokines, IL-1*β*, TNF-*α*, and IL-6/IL-10 ratio greatly increased in the hippocampus after the CUS. Paroxetine only reversed the increase of iNOS and TNF-*α* in the hippocampus (Figures 10(a) and 10(d)), whereas there was no effect on IL-6/IL-10 ratio and IL-1*β* (Figures 10(b) and 10(c)). HJDT (2 and 4 g/kg) treatment reversed the increase of iNOS, IL-6/IL-10, and TNF-*α* (Figures 10(a), 10(b), and 10(d)). Also HJDT 8 g/kg reduced the iNOS mRNA (Figure 10(a)), but there was no effect on the other mRNA of inflammatory cytokines.

### 3.11. HJDT Had No Effect on the 5-HT and DA Contents in the Hippocampus

**Figure 11:**
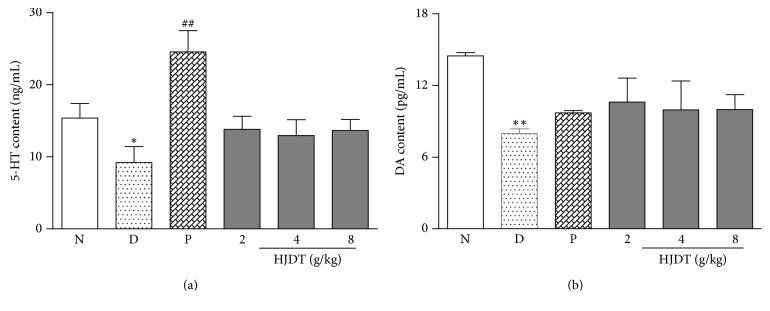
Effect of HJDT on the 5-HT and DA contents in the hippocampus after the CUS. The contents of 5-HT (a) and DA (b) decreased in CUS depression group. Paroxetine treatment only increased the 5-HT content (a) and there was no effect on DA content (b) in the hippocampus. There was no effect of HJDT on the contents of 5-HT and DA in the hippocampus (a, b). ^*∗*^*p* < 0.05 and ^*∗∗*^*p* < 0.01 relative to the normal control group; ^##^*p* < 0.01 relative to the CUS depression group. N: normal control group; D: CUS depression group; P: paroxetine group; HJDT 2: HJDT 2 g/kg group; HJDT 4: HJDT 4 g/kg group; HJDT 8: HJDT 8 g/kg group.

The results of ELISA showed the contents of 5-HT and DA both decreased in the CUS depression group when compared with the normal control group. Treatment with paroxetine reversed the decrease of 5-HT content (Figure 11(a)), but not DA content (Figure 11(b)) in the hippocampus. But HJDT (2, 4, and 8 g/kg) groups had no effects on the contents of 5-HT and DA in the hippocampus.

## 4. Discussion

The present study was aimed at examining the depression-like response following administration of aqueous extract of HJDT in rats which were subjected to the CUS. The major findings of this study include the fact that administration of HJDT during the CUS ameliorated depression-like behaviors, like anhedonia, decreased locomotor activity, despair condition, and cognitive function deficit, which could be relative to the inhibition of microglia activation, following with inflammation reaction inhibition and BDNF-TrkB-CREB pathway upregulation.

The stress-induced body weight decrease is an accompanying symptom of depression. However, HJDT did not gain the body weight decrease in CUS-treated rats except paroxetine treatment from day 35 to day 42 after the CUS, which is consistent with the previous findings about Traditional Chinese Medicine (TCM) treatment [19, 20]. The phenomenon of this change may be related to miss of the best period of growth and development in rats, which is an irreversible inhibition caused by the CUS. FST has been commonly used to evaluate the despair condition after stress and screen for potential antidepressants. Combined with no effects on body weight after HJDT treatment in our results, it suggested that the decreased immobility time in FST was unrelated with body weight. A reduction of sucrose consumption reflects anhedonia, which is one of the core symptoms of depression [21]. And this change could be restored by antidepressant treatment [22, 23]. The same results were found after HJDT treatment in FST. The decrease of total traveled distance, central distance ratio, and central time in OFT indicate a lower desire to explore, which may mimic psychomotor retardation [24]. Additionally, the low levels of rearing in OFT, and the increased immobility time in FST are often used as indices of a despair state [25, 26]. The rats exhibited improvement of depressive-like behaviors in OFT and in FST after HJDT or paroxetine treatments. The stress-induced cognitive decline is also an accompanying symptom of depression, which can be evaluated by Morris water maze task, the radial arm maze task [27, 28], and others. The NORT, less dependent on locomotion and reflecting both frontal cortical and hippocampal function, as well as a fear-motivated learning task, is often used in the cognitive impairment diseases [29, 30]. Our results showed the significant cognitive impairment in the CUS depression rats. HJDT (2 and 4 g/kg) and paroxetine increased the DR, DI, and the exploration time to novel object, which indicated the improvement of the cognitive performance.

The classic opinion on the development of depression considered that it was related to the monoamine neurotransmitter and serotonin (5-HT) system dysfunction [31, 32]. So at present the commonly used antidepressant drugs in clinic include selective 5-HT reuptake inhibitors, NA reuptake inhibitors, 5-HT and NA dual reuptake inhibitors. However, there was no effect of HJDT on the DA and 5-HT contents in our study, which suggested the improvement of HJDT on the depression-like behaviors may not involve the monoamine neurotransmitters.

Neuroinflammation is considered as one of the common pathogeneses of depression in recent research. The activation of the inflammatory immune system may induce the decrease of neurons regeneration and neurotrophic factors release and enhancement of the neuroinflammation reaction [33, 34]. Microglia are the major innate immune cell population in brain tissue and microglia-mediated inflammation is associated with the pathogenesis of various neuronal disorders, like depression, which are major cellular sources of TNF and IL-1 family of cytokines [35, 36]. Furthermore, several researches have reported that microglia cells in the brain were obviously activated in the brain of suicide victims [37], and antidepressants fluoxetine, paroxetine, and sertraline inhibited the microglial activation and reduced the release of inflammatory cytokines [38, 39]. Our study found that HJDT (2 and 4 g/kg) decreased the microglia number in the hippocampus, following a reduced production of proinflammatory cytokines, including iNOS, TNF-*α*, and IL-6/IL-10 ratio instead of IL-1*β*. iNOS is inflammation inducible, which has been considered as an important role in the depressive behavior induced by chronic stress exposure [40]. Recently, Peng et al. reported the iNOS inhibitor 1400 W exhibited a significant protection on neurons compared with CUS group [41]. TNF-*α*, IL-6, and IL-10 also played important roles in depression induced by stress, which caused the increase of proinflammation cytokines, including TNF-*α*, IL-6, and IL-1*β* in serum and hippocampus [24, 42]. Our results have confirmed that HJDT decreased the microglia number and the proinflammation cytokines concentration. But HJDT had no effect on the elevation of IL-1*β* after CUS, which was contrary with some reports [43, 44]. It may be related with the sensitivity of HJDT to different proinflammation cytokines.

In addition, activation of microglia can provoke the dysregulation of several growth factors in depression, like BDNF. BDNF and CREB had been reported to involve neuronal differentiation and survival as well as the synaptic plasticity associated with learning and memory in various nervous system disorders, including depression [45, 46]. It has been reported that chronic stress caused a downregulation of hippocampal BDNF or CREB levels and this reduction could be upregulated through antidepressant therapy [47, 48]. Our study found the expression of BDNF and its receptor TrkB as well as pCREB/CREB ratio decreased. It is worth mentioning that the pCREB DNA activity also decreased after CUS. Upregulation of the expression of BDNF-TrkB-CREB and the pCREB DNA activity in the hippocampus could contribute to the antidepressant effects of HJDT, manifested as improvement of depression-like behaviors.

## 5. Conclusion

In summary, the present study supports the notion that microglial activation, followed by inflammation reaction and BDNF decreasing, may contribute to the depressive-like behavior in a rat model in response to the CUS. HJDT treatment potentially ameliorated the depression-like behaviors. Additionally the research indicated that the effects of HJDT might be related to the decrease of microglia number, following with the reduction of inflammatory cytokines and the promotion of BDNF-TrkB-CREB pathway, which was independent of the HT and DA contents change. This antidepressant effect of HJDT was probably related to the synergistic effect of each active component, including berberine, baicalin, wogonoside, and gardenoside [8, 9].

## Supplementary Material

There are 8 components of HJDT, including geniposide, baicalin, palmatine, berberine, wogonside, baicalein and wogonin. The content of baicalin, berberine, wogonside and geniposide was higher than others.

## Figures and Tables

**Table 1:** Contents of active components in the decoction of HJDT (%) $(\overset{-}{x}\pm SEM$, *n* = 5).

	Geniposide	Baicalin	Palmatine hydrochloride	Berberine hydrochloride	Wogonoside	Baicalein	Wogonin
Content (%)	1.21 ± 0.02	5.23 ± 0.05	0.33 ± 0.01	4.25 ± 0.04	1.5 ± 0.01	0.45 ± 0.02	0.066 ± 0.02

*n*	5	5	5	5	5	5	5
