# Supplementary material for: Huanglian-Jie-Du-Tang Extract Ameliorates Depression-Like Behaviors through BDNF-TrkB-CREB Pathway in Rats with Chronic Unpredictable Stress
Source: Evid Based Complement Alternat Med. 2017 Jun 14;2017:7903918. doi: 10.1155/2017/7903918 (PMC5488320; doi:10.1155/2017/7903918)
Supplement: Supplementary file 1 — There are 8 components of HJDT, including geniposide, baicalin, palmatine, berberine, wogonside, baicalein and wogonin. The content of baicalin, berberine, wogonside and geniposide was higher than others. [file 7903918.f1.pdf]

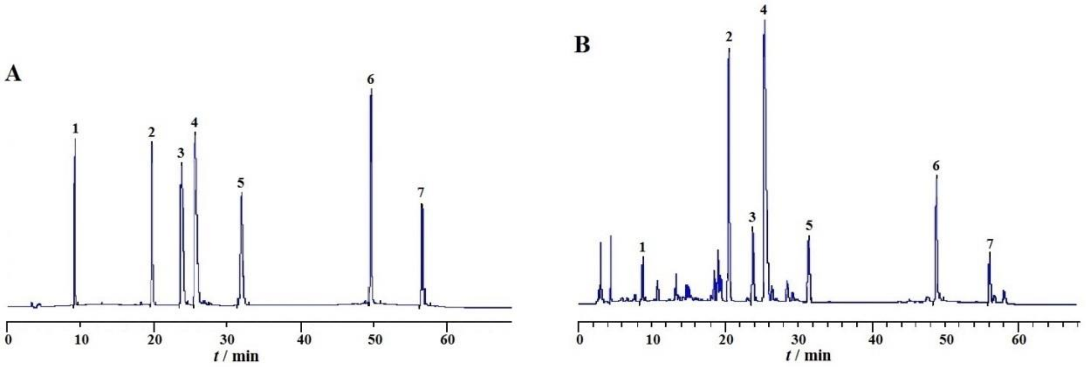

Fig. 1 The HPLC chromatograms of the active components in HJDT

1. Geniposide 2. Baicalin 3. Palmatine hydrochloride 4. Berberine hydrochloride 5. Wogonside 6. Baicalein 7. Wogonin

A: Standard products; B:HJDT
